# Multicomponent supervised tele-rehabilitation versus home-based self-rehabilitation management after anterior cruciate ligament reconstruction: a study protocol for a randomized controlled trial

**DOI:** 10.1186/s13018-024-04871-0

**Published:** 2024-06-28

**Authors:** Kexin Wang, Linbo Peng, Mingke You, Qian Deng, Jian Li

**Affiliations:** 1 Department of Clinical Research Management, West China Hospital, Sichuan University, Chengdu, China; 2 Sports Medicine Center, West China Hospital, Sichuan University, Chengdu, China; 3 Department of Orthopedics and Orthopedic Research Institute, West China Hospital, Sichuan University, Chengdu, China

**Keywords:** Anterior cruciate ligament, ACL reconstruction, Tele-rehabilitation

## Abstract

**Introduction:**

Our study aims to assess the effectiveness of multicomponent supervised tele-rehabilitation compared to home-based self-rehabilitation management in patients following anterior cruciate ligament reconstruction (ACLR).

**Methods:**

The current study is designed as a single-center, single-blinded, randomized controlled, two-arm trial. Participants will be randomized and allocated at a 1:1 ratio into either a multicomponent supervised tele-rehabilitation group or a home-based self-rehabilitation group. All participants receive uniform preoperative education through the HJT software. Participants in the intervention group undergo multicomponent supervised tele-rehabilitation, while those in the control group follow a home-based self-rehabilitation program. All the participants were assessed and measured for the included outcomes at the outpatient clinic before the procedure, and in 2, 4, 8, 12, and 24 weeks after ACLR by two assessors. The primary outcome was the percentage of patients who achieve a satisfactory active ROM at the 12 weeks following the ACLR. The satisfactory active ROM was also collected at 2, 4, 8, and 24 weeks after ACLR. The secondary outcomes were active and passive range of motion (ROM), pain, muscle strength, and function results.

**Registration details:**

Ethical approval has been obtained from the West China Hospital Ethics Committee (approval number 2023−1929, December 2023). The trial has been registered on ClinicalTrials.gov (registration number NCT06232824, January 2024).

**Supplementary Information:**

The online version contains supplementary material available at 10.1186/s13018-024-04871-0.

## Introduction

Anterior cruciate ligament (ACL) rupture is one of the most common orthopedic injuries, with a yearly incidence of 0.03% [1]. Due to the instability of the knee caused by ACL rupture, ACL reconstruction (ACLR) is often necessary and has become a frequently performed orthopedic surgery [2]. Returning to daily activities and sports is a primary goal of ACLR [3]. However, within the first postoperative year following surgery, two-thirds of patients have not returned to their pre-injury level [4]. A more extended and effective postoperative rehabilitation may be needed compared to what is typically advocated after ACLR surgery. Several important clinical practice guidelines recommend postoperative rehabilitation following ACLR to restore muscle strength and function, alleviate pain and symptoms, and facilitate a successful return to pre-injury sporting activities [5].

In a randomized controlled trial (RCT), Grant et al. determined that a minimally supervised home rehabilitation program was more effective in achieving an acceptable range of motion (ROM) than standard physical therapy in the initial 3 months after ACLR [6]. A more extended follow-up of 38 months revealed that the home-based rehabilitation exhibited a significantly higher ACL Quality of Life (QoL) score compared to the physical therapy-supervised group, indicating that the supervised home rehabilitation program was both cost-effective and beneficial for future rehabilitation practices [7]. A recent meta-analysis corroborated these findings, suggesting that standard physical therapy did not yield superior outcomes when compared to supervised home rehabilitation in patients undergoing ACLR [8]. However, it is important to note that these studies do not advocate for the complete abandonment of professional supervision in ACLR rehabilitation. Rather, they emphasize that the frequency of supervision may not be as crucial as the patient’s understanding of the rehabilitation process and their adherence to the prescribed program [9].

With advancements in telecommunication networks and tele-applications, healthcare professionals, including surgeons and rehabilitation therapists, can now remotely evaluate, educate, diagnose, and even treat patients using information and communication technologies [10]. Considering the challenges posed by the COVID-19 pandemic, transitioning to tele-rehabilitation has emerged as a viable option [11]. Studies have shown that tele-rehabilitation yields non-inferior outcomes compared to traditional face-to-face rehabilitation for various musculoskeletal disorders [12, 13]. Guo et al. investigated the impact of mobile health-based home rehabilitation education and found that this intervention led to improvements in knee function, muscle atrophy, and joint pain compared to home-based rehabilitation exercises alone, as early as 6 weeks post ACLR [14]. Additionally, a randomized controlled trial (RCT) demonstrated that mobile applications reduced the need for in-person visits during the initial 6 weeks following ACLR, resulting in cost savings for both patients and the healthcare system [15]. It is worth noting, however, that while these studies utilize mobile devices and software for patient follow-up and evaluation, they may lack a visual user-interface guidance for functional exercises [14, 15].

After being discharged from the hospital, most patients adhere to the prescribed rehabilitation schedule outlined by surgeons and rehabilitation therapists [16]. However, they often face limitations, as they can only seek guidance by returning to the hospital in case of exercise-related queries. This approach is not only inefficient but also challenging to sustain, potentially leading to a non-standardized rehabilitation program and undermining patients’ understanding and adherence to the prescribed regimen.

To address these issues, we have developed an individualized multicomponent supervised tele-rehabilitation management application. This innovative tool allows us to provide education, visual user-interface guidance for functional exercises, interactive real-time communication, and evaluation throughout perioperative and postoperative follow-up periods. Therefore, our study aims to assess the effectiveness of multicomponent supervised tele-rehabilitation compared to home-based self-rehabilitation in patients following ACLR. We hypothesize that multicomponent supervised tele-rehabilitation will demonstrate superior effects over home-based self-rehabilitation in terms of ROM, pain, muscle strength, and overall function.

## Methods

### Study design

**Fig. 1:**
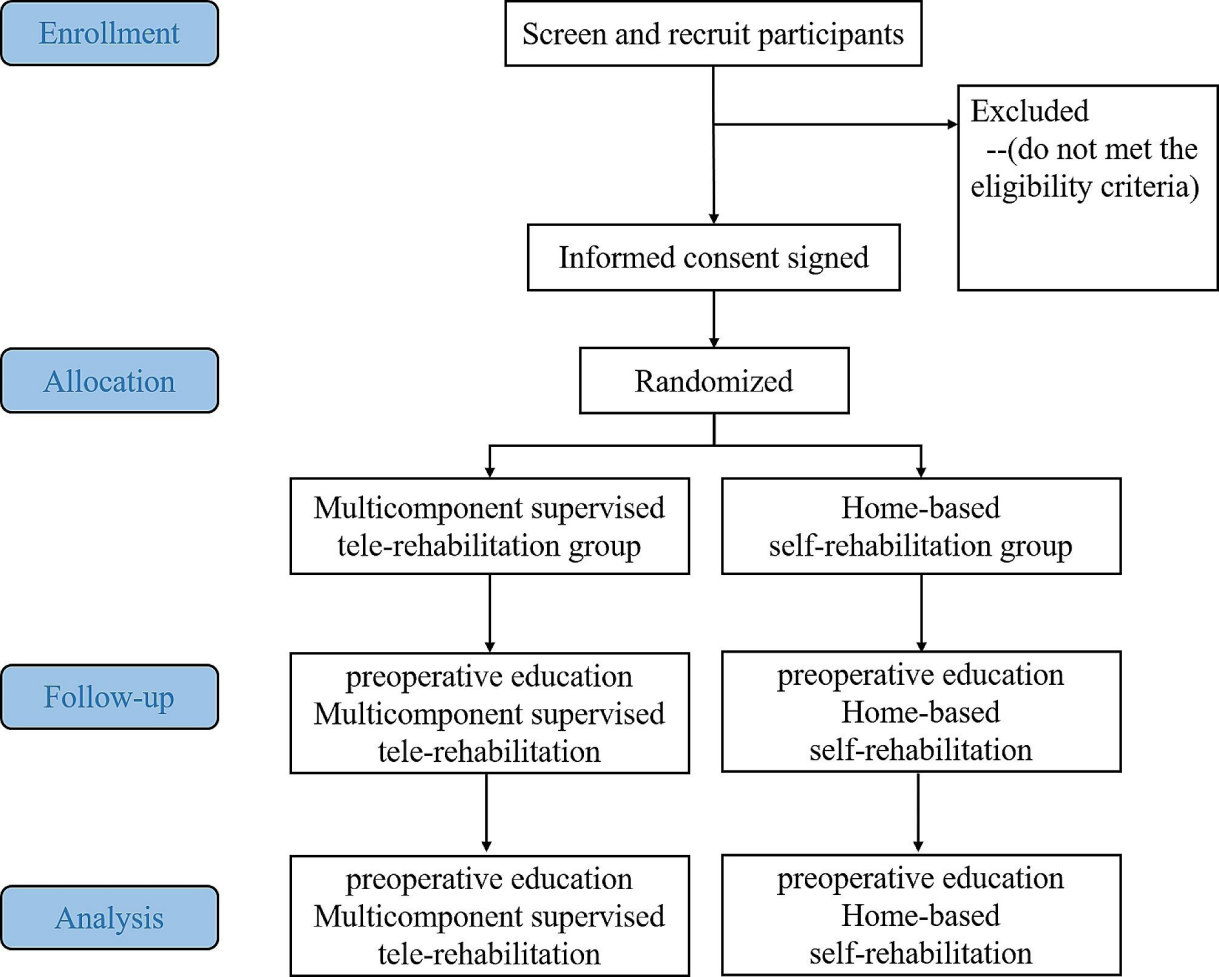
The flowchart of the whole study

The current study is designed as a single-center, single-blinded, randomized controlled, two-arm trial, with the overall flowchart depicted in Fig. 1. We intend to allocate participants in a 1:1 ratio to compare multicomponent supervised tele-rehabilitation versus home-based self-rehabilitation. Ethical approval has been obtained from the West China Hospital Ethics Committee (approval number 2023−1929, December 2023). The trial has been registered on ClinicalTrials.gov (registration number NCT06232824, January 2024). Each participant is required to provide informed consent before enrolling in the study. The protocol adheres to the 2013 Standard Protocol Items: Recommendations for Intervention Trials guidelines.

### Participants

We screen and recruit participants from the department of orthopaedic surgery and sports medicine, West China Hospital, Sichuan University in China from July 2024 to July 2025. This study will only recruit patients who meet the following criteria:1. Aged between 18 and 50 years at the time of recruit; 2. BMI between 16 and 28 kg/m²; 3. acute unilateral ACL rupture; 4. plan for an ACLR surgery (with autologous hamstrings tendon reconstruction) under arthroscopy; 5. ACL rupture to ACLR within 3 months; 6. Patients can independently use mobile software and HJT software under the guidance of staff.

The exclusion criteria of this study include: (1) With synthetic tendon reconstruction; (2) Concomitant meniscus lesion which needs operation; (3) Concomitant other ligaments injury which needs operation; (4) Concomitant intra-articular knee fracture; (5) Concomitant fracture or injury which may affect postoperative exercise; (6) Previous history of knee infection, fracture, and surgery; (7) Participate in knee exercises and/or rehabilitation programs in the past three months; (8) Living outside the city, regular return to the hospital for follow-up cannot be guaranteed; (9) Serious cardiopulmonary disease and unable to participate in rehabilitation exercise; (10) Other reasons for exclusion (mental disorders, stroke, pregnancy, etc.).

### Sample size determination

We use PASS software to calculate the sample size. We set the percentage of patients who achieve a satisfactory active ROM at the 3 months following the ACLR as the primary outcome. It was reported that approximately 95% of patients should achieve an acceptable ROM at 3 months after ACLR [17]. Besides, An estimated 20% difference was reported to be clinically significant [6]. To achieve a power of 80% (α = 0.05), a minimum of 44 subjects is required for each group. Taking a 20% attrition rate into consideration, a total of 110 patients (55 per group) will be recruited in the study.

### Randomization procedure and blinding

We use R software to generate a random sequence of 110 numbers, which were placed into opaque sealed envelopes by an independent researcher to avoid selection bias. Participants will be randomized and allocated at a 1:1 ratio into either a multicomponent supervised tele-rehabilitation group or a home-based self-rehabilitation group. Given the particularity of postoperative rehabilitation, it is hard to blind the participants. Therefore, blinded participants were not implemented in our study. All the participants received the education and rehabilitation program on the same mobile phone application. Before the intervention, an independent researcher communicated with the participants to inform the method of using the mobile application. Apart from the mobile application, no paper rehabilitation program materials will be distributed to participants. Admission, ACLR surgery, follow-up, and assessment of all participants were conducted separately and will not be arranged in the same ward room to avoid discussion and communication between participants. The ACLR surgery was performed by a senior surgeon who was blinded to the group allocation. In addition, we selected two assessors who were blinded to the group allocation to measure the baseline data and follow-up outcomes after the intervention. The collection and analysis of data were carried out by two independent researchers who were also blinded to the group allocation.

### Experimental procedure

**Fig. 2:**
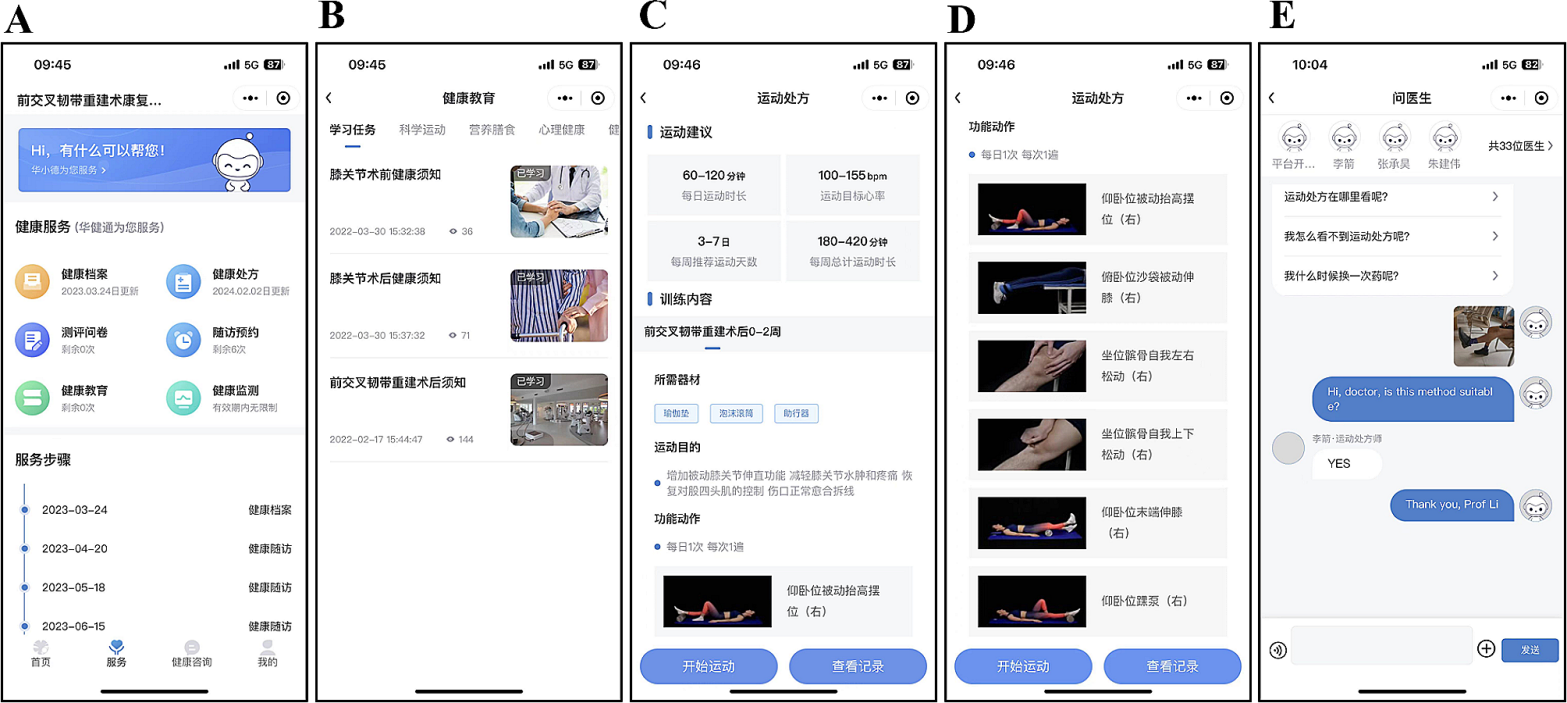
The interface of the HJT software. **A**: The main page of the HJT software, the timeline of the experiment was also presented in the main page. **B**: The page of the software displays the preoperative education. **C-D**: The page of the software displays the part of postoperative rehabilitation. **E**: The page of the software displays the interactive real-time communication

The entire program comprises preoperative education and postoperative rehabilitation, both within and outside the hospital setting. All participants receive uniform preoperative education through the HJT software. Participants in the intervention group undergo multicomponent supervised tele-rehabilitation, while those in the control group follow a home-based self-rehabilitation program. The postoperative rehabilitation protocols for both groups are presented and implemented through the HJT software. The classical interface of the HJT software was showen in Fig. 2.

### Intervention

Participants in the intervention group could access the rehabilitation content applicable to their current phase each day and confirm their willingness to execute it through the application. Additionally, participants have the capability to communicate with therapists via the mobile phone application, enabling them to send text, voice messages, images, and videos throughout the entire experiment. The specific rehabilitation protocol is developed based on the best available evidence for ACLR and is discussed with experienced surgeons and physiotherapists in our center [5, 18–20]. Participants in the intervention group receive comprehensive education and rehabilitation program through the mobile phone application, which includes text, photos, and videos. On the first day of enrollment, the doctor informs participants about the importance of rehabilitation and guides them on how to use the mobile phone application. The postoperative rehabilitation protocol is organized into five phases: Phase 1 (0–2 weeks), Phase 2 (3–4 weeks), Phase 3 (5–8 weeks), Phase 4 (9–12 weeks), and Phase 5 (after 13 weeks).

### Phase 1 (0–2 weeks)

The objectives of this phase are as follows: (1) Protect the graft; (2) Reduce knee joint edema and pain; (3) Restore patellar mobility; (4) Restore knee passive extension; (5) Improve knee joint flexion; (6) Restore the strength of the quadriceps femoris muscle; (7) Promote wound healing and remove sutures.

The exercise items for this phase include: (1) Passive straightening of the knee joint (with/without sandbag aid); (2) Alternating placement in straight and training positions; (3) Knee joint training position; (4) Ankle pump; (5) Straight leg raise; (6) Lying prone and lifting the leg (with/without elastic band aid); (7) Lying on the side and lifting the leg (with/without elastic band aid); (8) Isometric contraction of the quadriceps femoris muscle; (9) Isometric contraction of the hamstring muscle in the seated position; (10) Partially loaded walking (double walking stick aid).

### Phase 2 (3–4 weeks)

The objectives of this phase are as follows: (1) Fully restore knee ROM in both extension and flexion; (2) Further enhance lower limb muscle strength; (3) Improve proprioception, balance, and neuromuscular coordination; (4) Restore normal gait.

The exercise items for this phase include those from Phase 1, plus: (1) Active knee joint exercises (range 0-120°); (2) Removal of wound sutures and scar massage; (3) Gravity transfer training; (4) Walking exercise without a walking stick; (5) Lunge squat with a stick; (6) Side lunge with armchair support.

### Phase 3 (5–8 weeks)

The objectives of this phase are as follows: (1) Further enhance lower limb muscle strength; (2) Achieve full restoration of knee joint ROM; (3) Improve proprioception, balance, and neuromuscular coordination.

The exercise items for this phase include: (1) Passive flexion of the knee joint (with sandbag aid); (2) Alternating placement in straight and training positions; (3) Ankle pump; (4) Lying prone and lifting the leg (with elastic band aid); (5) Lying on the side and lifting the leg (with elastic band aid); (6) Knee joint active exercises without load(range 0-120°); (7) Prone position active knee bending; (8) Scar massage; (9) Stride stretch (front and back); (10) Walking exercise without a walking stick to correct gait; 11. Step forward and backward stretching; 12. Standing balance and gravity transfer using a balance board; 13. Gait exercises (8-step, S-step, turn back step).

### Phase 4 (9–12 weeks)

The objectives of this phase include: (1) Restore symmetrical active joint ROM fully; (2) Engage in higher levels of neuromuscular control activities and start jogging on plastic tracks or soft surfaces.

The exercise items of this phase include:1. Prone position active knee bending;2. Double leg glute bridge; 3. Bedside kneeling position progressive knee bend; 4. Scar massage; 5.Stand on one leg; 6. Stepping exercises (back and forth, sideways stepping exercises); 7. Stride stretch (front and back); 8. Gait exercise (8-step, S-step, turn back step);9. Speed-walking for 15 min; 10. Consecutive ten single-leg squats to 60°; 11. Jogging for 5–10 min; 12. Jump forward on one foot.

### Phase 5 (after 13 weeks)

The objectives of this phase include: (1) Increase muscle strength; (2) Increase proprioception.

The exercise items of this phase include: (1) Bedside kneeling position progressive knee bend; (2) Single leg glute bridge; (3) Stand on one leg; (4) Stepping exercises (back and forth, sideways stepping exercises); (5) Stride stretch (front and back); (6) Consecutive ten single-leg squats to 60°;7. Jogging for 5–10 min; 8. Alternate sideways walk; 9. Alternate side jumps; 10. Walk backwards; 11. Skipping rope; 12. Gait exercise (8-step, S-step, turn back step);13. Single leg jump up and down stairs.

At the 2 weeks, 4 weeks, 8 weeks, 12 weeks, and 6 months after ACLR, all participants went to the outpatient clinic for follow-up by physiotherapist. If participants can complete the exercise items in this phase and achieve the objectives of this phase, they will be switch to the next phase rehabilitation and provide face-to-face guidance for exercise methods.

### Control

Participants in the control group could only receive a graphic and textual minimal postoperative rehabilitation plan on the mobile phone application. However, the participants were not informed the frequency and intensity of the rehabilitation items. They could not communicate with therapists online. Participants in the control group were expected to exercise unsupervised postoperatively.

At the 2, 4, 8, 12, and 24 weeks after ACLR, all participants went to the outpatient clinic for follow-up by physiotherapist to provide face-to-face guidance for exercise methods. Physiotherapist would clarify the content of the rehabilitation plan if any doubt, but will not provide information extending the prearranged scope.

### Outcome measures

All the participants were assessed and measured for the included outcomes at the outpatient clinic before the procedure, and in 2, 4, 8, 12, and 24 weeks after ACLR by two assessors. Outcome measures were conducted before face-to-face guidance for exercise methods at every visit in the outpatient clinic.

### Demographic characteristics

Participant characteristics (including age, sex, body mass index (BMI), comorbidities, physically active (professional, amateur, or recreational athletes), and history of smoking) will be collected 1 week before the trial begins. Surgical details (including date, surgical side, American Society of Anesthesiologists (ASA) score, graft type, and concomitant injuries/procedures) will be recorded from the operation document after discharge.

### Primary outcome

The primary outcome was the percentage of patients who achieve a satisfactory active ROM at the 12 weeks following the ACLR. In the first 3 months after ACLR, the achievement of acceptable knee active extension and flexion was regarded as what matters most for a successful recovery. A good knee active ROM could guarantee an expectedly continue improvement [6]. The satisfactory active ROM was also collected at 2, 4, 8, and 24 weeks after ACLR.

### Secondary outcomes

The secondary outcomes were active and passive ROM, pain, muscle strength, and function results.

### ROM

The active and passive ROM (flexion and extension) of the affected side knee were measured with goniometry [6, 21].

### Pain

The postoperative pain was measured with visual analogue scale (VAS). The VAS scale ranges from 0 to 10 points, 0 points represent no pain, while 10 points represent the worst imaginable pain [22, 23].

### Muscle strength

The isokinetic concentric strength test at 90°/s was used to assess quadriceps and hamstring muscle strength. Isokinetic concentric extenso was the best rated with sufficient intrarater reliability and construct validity for the measurement of knee muscle strength [24].

### Function

The self-reported knee-specific function was represented by 2000 International Knee Documentation Committee (IKDC) subjective knee form, which was widely used to access knee symptoms and functions after ACLR [25]. Besides, the knee injury and osteoarthritis outcome score (KOOS), the Tegner activity scale, and the Lysholm knee scoring scale were also used to assess functional outcomes [23, 26].

### Statistical analysis

All the data collect and analyses will be performed by two independent researchers who were blinded to the group allocation in SPSS 22.0 (IBM, USA) software. Participants who dropout or lost to follow-up would be excluded. Demographic characteristics and follow-up outcome data would be included in the analysis. The independent sample t-test or nonparametric analysis would be used to evaluate continuous variables, while chi-square test or Fisher’s exact test evaluate the categorical variables, and repeated-measures ANOVA evaluate the repeated measures data.Bilateral test was utilized in all the analysis and *P* < 0.05 was regarded as statistically significant.

### Ethics and dissemination

The study was approved by the ethics committee of West China Hospital of Sichuan University (approval number 2023−1929, Dec 2023). Before participate recruitment, the study was registered in the ClinicalTrials.gov (registration number NCT06232824, Jan 2024). This study complies with the Declaration of Helsinki and written informed consent would obtained from each participant prior to enrolment. Any important protocol amendments will be reported to the ethics committees and ClinicalTrials.gov. The study findings would be disseminated in scientific forums, peer-reviewed publications, and international conferences. Outcomes would be propagated regardless of the magnitude or direction of the impact.

## Discussion

The results of the study may demonstrate that multicomponent supervised tele-rehabilitation could accelerate the recovery over traditional home-based self-rehabilitation following ACLR. Multicomponent supervised tele-rehabilitation is expected to relieve pain, improve ROM, muscle strength, and function.

The health care system has undergone significant changes due to the profound effects of the COVID-19 pandemic [27]. The inaccessibility of face-to-face post-operative rehabilitation during this period has prompted the swift emergence of contactless rehabilitation methods [28].Supervised rehabilitation under the guidance of a licensed therapist might prove more efficacious than engaging in unsupervised exercise [29]. A retrospective cohort study found that telerehabilitation after ACLR seems to provide a superior short-term outcome compared to hospital-based rehabilitation during the COVID-19 pandemic [30].

The purpose of ACLR is to maximize the stability and functional capacity of the knee joint, and therefore facilitate return to sports [4]. However, approximately 50% of individuals with injuries will manifest symptomatic osteoarthritis within a decade, irrespective of whether they undergo operative or non-operative treatment [31]. Despite the perceived benefits of exercise therapy for ACLR, there is a lack of consensus on its optimal components [32]. Most trials conducted after ACL injury lack individualized interventions, employing the same program for all patients, and fail to adequately document exercise frequency, intensity, volume, and progression [33]. Therefore, implementing personalized and supervised ACLR rehabilitation is crucial. Technological advancements and digital solutions may be considered promising tools for implementing a comprehensive rehabilitation approach for patients after ACLR. Recent studies have also confirmed the potential advantages of remote rehabilitation in post-ACLR management [34]. In the treatment of ACL injuries, rehabilitation assumes a pivotal role, yet its implementation prior to or following ACLR have not be standardized in a uniform manner due to the intricate nature of individual cases in the context of modern evidence [35–42].Unlike many tele-rehabilitation programs that primarily focus on post-discharge follow-up, weconducted a multi-component supervised tele-rehabilitation with comprehensive services. We offer education, visually guided user interface assistance for functional exercises, interactive real-time communication, and evaluations during both active and passive follow-up periods, which offering additional evidence supporting a advanced tele-rehabilitation after ACLR.

Figure 1. The flowchart of the whole study.

Figure 2. The interface of the HJT software. **A**: The main page of the HJT software, the timeline of the experiment was also presented in the main page. **B**: The page of the software displays the preoperative education. **C-D**: The page of the software displays the part of postoperative rehabilitation. **E**: The page of the software displays the interactive real-time communication.

### Electronic supplementary material

Below is the link to the electronic supplementary material.

Supplementary Material 1

## Data Availability

No datasets were generated or analysed during the current study.
