# Supplementary material for: Multicomponent supervised tele-rehabilitation versus home-based self-rehabilitation management after anterior cruciate ligament reconstruction: a study protocol for a randomized controlled trial
Source: J Orthop Surg Res. 2024 Jun 28;19:381. doi: 10.1186/s13018-024-04871-0 (PMC11212401; doi:10.1186/s13018-024-04871-0)

**The postoperative rehabilitation protocol in the intervention group is organized into five phases: Phase 1 (0-2 weeks), Phase 2 (3-4 weeks), Phase 3 (5-8 weeks), Phase 4 (9-12 weeks), and Phase 5 (after 13 weeks).**

**Phase 1 (0-2 weeks)**

**Objectives:**

1. Protect the graft;

2. Reduce knee joint edema and pain;

3. Restore patellar mobility;

4. Restore knee passive extension;

5. Improve knee joint flexion;

6. Restore the strength of the quadriceps femoris muscle;

7. Promote wound healing and remove sutures.

**Exercise items**

1. **Passive straightening of the knee joint (with/without sandbag aid)**

10secends/ time，10 times /group，3-5groups/day


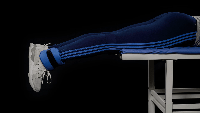


1. **Alternating placement in straight and training positions**

10-15 minutes/ time in the training position, 3-5 times a day


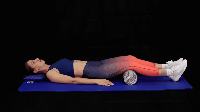


1. **Knee joint training position**

10-15 minutes/time, 3-5 times a day


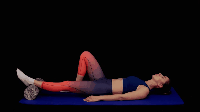


1. **Ankle pump**

10 times/group, 10 groups/day


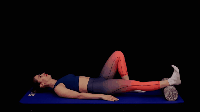


1. **Straight leg raise**

10 seconds/ time，10times/group，3-5 groups/ cohort, 2 cohorts/ day


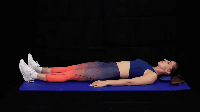


1. **Lying prone and lifting the leg (with/without elastic band aid)**

10times/ group，3-5 groups/ cohort，1 cohort/ day


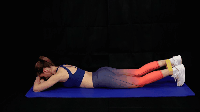


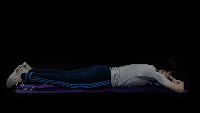


1. **Lying on the side and lifting the leg (with/without elastic band aid)**

10times/ group，3-5 groups/ cohort，1 cohort/ day


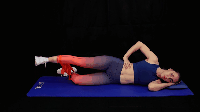


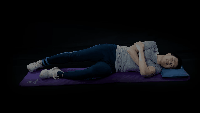


1. **Isometric contraction of the quadriceps femoris muscle**

10times/ group，3-5 groups/ cohort，2 cohorts/ day


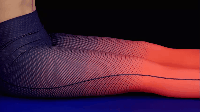


1. **Isometric contraction of the hamstring muscle in the seated position**

10times/ group，3-5 groups/ cohort，2 cohorts/ day


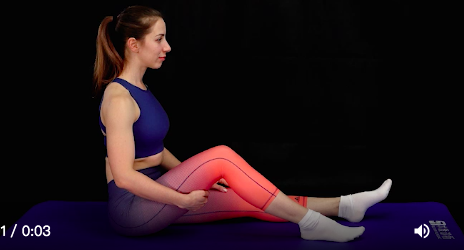


1. **Partially loaded walking (double walking stick aid)**

3-5min/time, 1 time/ day


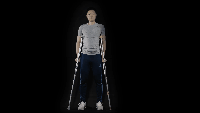


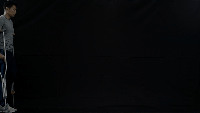


**Phase 2 (3-4 weeks)**

**Ojectives:**

1. Fully restore knee ROM in both extension and flexion;

2. Further enhance lower limb muscle strength;

3. Improve proprioception, balance, and neuromuscular coordination;

4. Restore normal gait.

**Exercise items**

**Phase 1 plus:**

1. **Active knee joint exercises (range 0-120°)**

5 times/ group，3 groups/ cohort，2 cohorts/ day


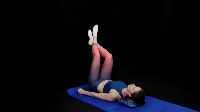


1. **Removal of wound sutures and scar massage**

3 times/ group，3 groups/ cohort，1 cohort/ day


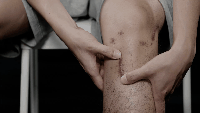


1. **Gravity transfer training**

10 times/ group，3-5 groups/ cohort，1 cohort/ day


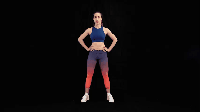


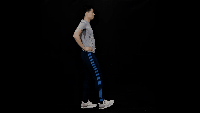


1. **Walking exercise without a walking stick**

3 times/ group，3 groups/ cohort，1 cohort/ day


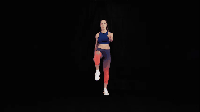


1. **Lunge squat with a stick**

12 times/ group，3 groups/ cohort，1 cohort/ day


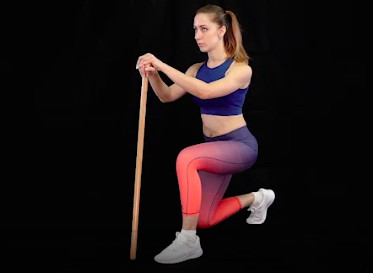


1. **Side lunge with armchair support**

12 times/ group，3 groups/ cohort，1 cohort/ day


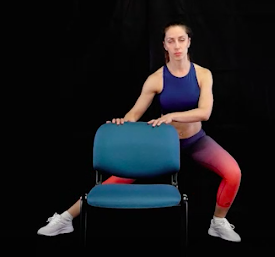


**Phase 3 (5-8 weeks)**

**Objectives:**

1. Further enhance lower limb muscle strength;

2. Achieve full restoration of knee joint ROM;

3. Improve proprioception, balance, and neuromuscular coordination.

**Exercise items：**

1. **Passive flexion of the knee joint (with sandbag aid)**

10 times/group, 3-5 groups/ day


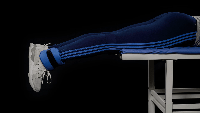


1. **Alternating placement in straight and training positions**

10-15min/time，3-5 groups/ day


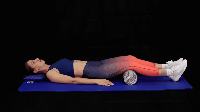


1. **Ankle pump**

10 times/group, 10 groups/ day


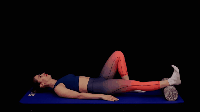


1. **Lying prone and lifting the leg (with elastic band aid)**

10 times/ group，3-5 groups/ cohort，1 cohort/ day


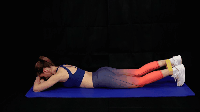


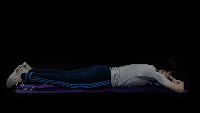


1. **Lying on the side and lifting the leg (with elastic band aid)**

10 times/ group，3-5 groups/ cohort，1 cohort/ day


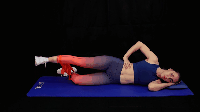


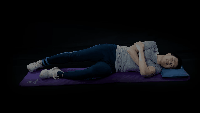


1. **Knee joint active exercises without load(range 0-120°)**

5 times/ group，3 groups/ cohort，2 cohorts/ day


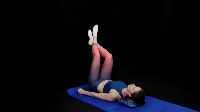


**7. Prone position active knee bending**

12 times/ group，3 groups/ cohort，1 cohort/ day


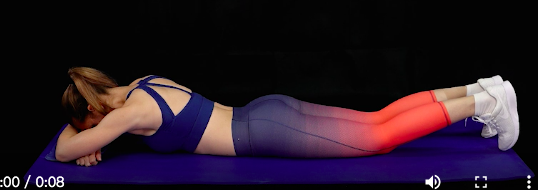


**8. Scar massage**

60 seconds/time, 3 times/ group, 3 group /cohort, 1 cohort/ day


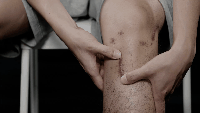


**9. Stride stretch (front and back)**

12 times/ group, 3 group /cohort, 1 cohort/ day


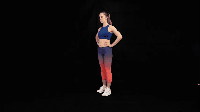


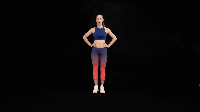


**10. Walking exercise without a walking stick to correct gait**

5-10 min/ day


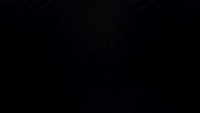


1. **Step forward and backward stretching**

15 times/ group, 3 group /cohort, 1 cohort/ day


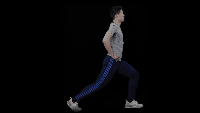


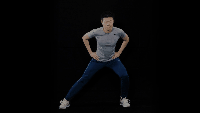


**12. Standing balance and gravity transfer using a balance board**

5–10 min/day


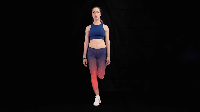


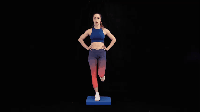


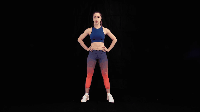


**13. Gait exercises (8-step, S-step, turn back step)**

5–10 min


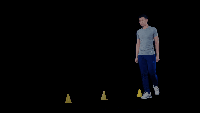


**Phase 4 (9-12 weeks)**

**Objectives:**

1. Restore symmetrical active joint ROM fully;

2. Engage in higher levels of neuromuscular control activities and start jogging on plastic tracks or soft surfaces.

**Exercise items**

1. **Prone position active knee bending**

12 times/ group, 3 group /cohort, 1 cohort/ day


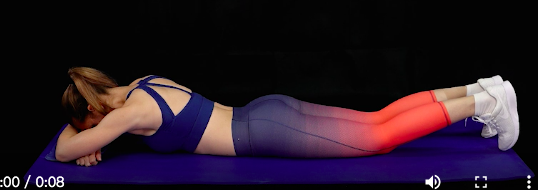


1. **Double leg glute bridge**
2. times/ group, 4 group /cohort, 1 cohort/ day


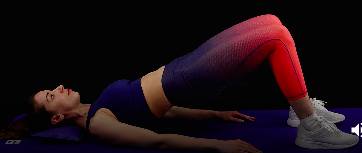


1. **Bedside kneeling position progressive knee bend**

60 seconds/ times, 3 times/ group, 3 group /cohort, 2 cohorts/ day


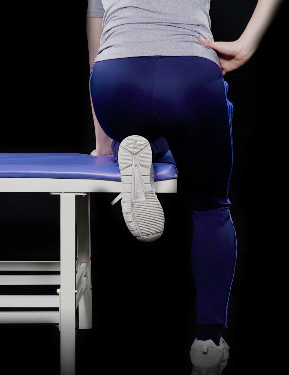


1. **Scar massage**

60 seconds/ times, 3 times/ group, 3 group /cohort, 1 cohort/ day


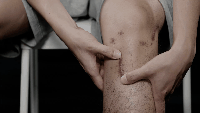


1. **Stand on one leg**

5-10 min


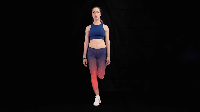


1. **Stepping exercises (back and forth, sideways stepping exercises)**

12 times/ group, 3 group /cohort, 1 cohort/ day


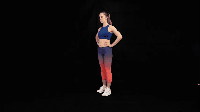


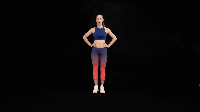


1. **Stride stretch (front and back)**

10 seconds/ times, 15 times/ group, 3-5 group /cohort, 1 cohort/ day


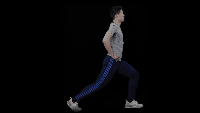


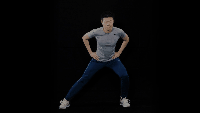


**8. Gait exercise (8-step, S-step, turn back step)**

5–10 min


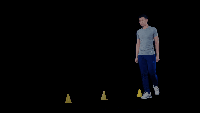


**9. Speed-walking for 15 min**

15 min


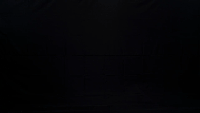


**10. Consecutive ten single-leg squats to 60°**

12 times/ group, 3 group /cohort, 1 cohort/ day


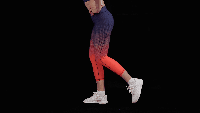


**11. Jogging for 5-10 min**

5-10 min


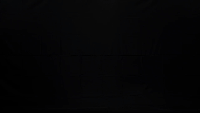


**12. Jump forward on one foot**

5-10 times/ group, 3-5 group /cohort, 1 cohort/ day


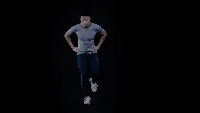


**Phase 5 (after 13 weeks)**

**Objectives:**

1. Increase muscle strength;

2. Increase proprioception.

**Exercise items:**

**1. Bedside kneeling position progressive knee bend**

60 seconds/ times, 3 times/ group, 3 group /cohort, 2 cohorts/ day


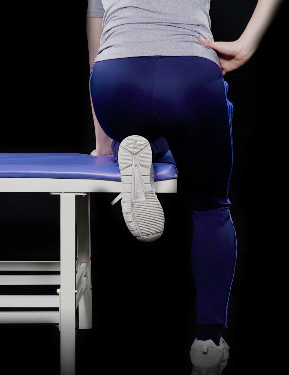


**2. Single leg glute bridge**

12 times/ group, 4 group /cohort, 1 cohort/ day


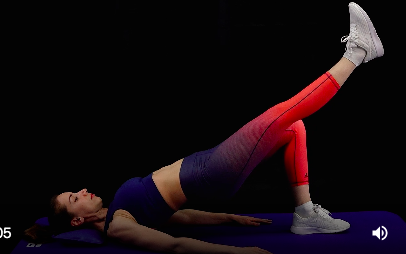


**3. Stand on one leg**

5-10 min


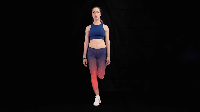


**4. Stepping exercises (back and forth, sideways stepping exercises)**

12 times/ group, 3 group /cohort, 1 cohort/ day


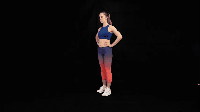


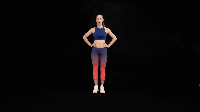


**5. Stride stretch (front and back)**

10 seconds/ times, 15 times/ group, 3-5 group /cohort, 1 cohort/ day


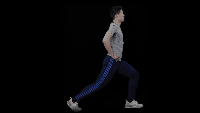


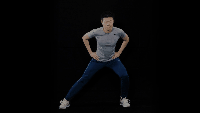


**6.Consecutive ten single-leg squats to 60°**

12 times/ group, 3 group /cohort, 1 cohort/ day


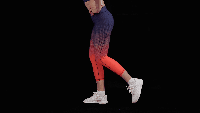


**7. Jogging for 5-10 min**

5-10 min


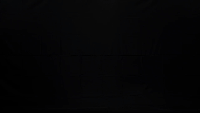


**8. Alternate sideways walk**

5-10 min/ group, 3-5 group /cohort, 1 cohort/ day


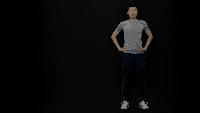


**9. Alternate side jumps**

5-10 min/ group, 3-5 group /cohort, 1 cohort/ day


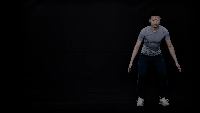


**10. Walk backwards**

5-10 min/ group, 3-5 group /cohort, 1 cohort/ day


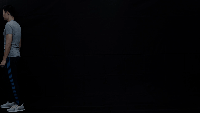


**11. Skipping rope**

5-10 min/ group, 3-5 group /cohort, 1 cohort/ day


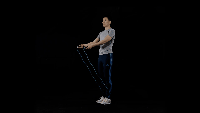


**12. Gait exercise (8-step, S-step, turn back step)**

5–10 min


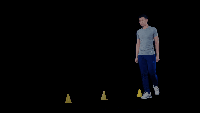


**13. Single leg jump up and down stairs**

12 times/ group, 3 group /cohort, 1 cohort/ day


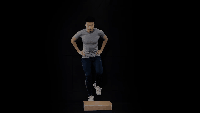


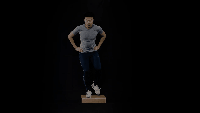

Supplement: Supplementary file 1 — Supplementary Material 1 [file 13018_2024_4871_MOESM1_ESM.docx]
